# Tumor Microenvironment–Responsive Peptide-Based Supramolecular Drug Delivery System

**DOI:** 10.3389/fchem.2020.00549

**Published:** 2020-07-22

**Authors:** Wenbo Zhang, Lanlan Yu, Tianjiao Ji, Chenxuan Wang

**Affiliations:** ^1^State Key Laboratory of Medical Molecular Biology, Department of Biophysics and Structural Biology, Peking Union Medical College, Institute of Basic Medical Sciences, Chinese Academy of Medical Sciences, Beijing, China; ^2^Laboratory for Biomaterials and Drug Delivery, Department of Anesthesiology, Boston Children's Hospital, Harvard Medical School, Boston, MA, United States

**Keywords:** peptide, supramolecular assembly, stimuli–response, drug delivery, tumor microenvironment

## Abstract

Physical and biochemical differences between tumor tissues and normal tissues provide promising triggering factors that can be utilized to engineer stimuli-responsive drug delivery platforms for cancer treatment. Rationally designed peptide-based supramolecular architectures can perform structural conversion by responding to the tumor microenvironment and achieve the controlled release of antitumor drugs. This mini review summarizes recent approaches for designing internal trigger-responsive drug delivery platforms using peptide-based materials. Peptide assemblies that exhibit a stimuli-responsive structural conversion upon acidic pH, high temperature, high oxidative potential, and the overexpressed proteins in tumor tissues are emphatically introduced. We also discuss the challenges of current peptide-based supramolecular delivery platforms against cancer.

## Introduction

The clinical efficacy and outcome of conventional molecular chemotherapeutics against tumors are limited by several undesirable properties, including the poor solubility, the short half-life *in vivo*, the weak penetration capability, and the low specificity (Tang et al., 2014; Liu et al., 2016; Wang et al., 2017, 2018; Luo et al., 2019). To overcome these drawbacks of conventional cancer chemotherapeutics, various nanoparticle-based drug delivery systems (DDSs) have been developed (Versluis et al., 2016; Gerbelli et al., 2019; Xiao et al., 2019b). Ingeniously designed DDSs can improve the bioavailability of drugs and/or minimize the adverse effects of drugs on normal tissues (Liu et al., 2016; Wang et al., 2017). Currently, there are a diverse range of nanoscale carriers to meet different practical requirements, including inorganic nanoparticles (e.g., silica nanoparticles, quantum dots, gold nanoparticles, carbon-based, and magnetic iron oxide–based nanostructures), synthetic organic nanoparticles (e.g., polymer-based nanostructures and dendrimers), and bio-original nanomaterials (e.g., lipid-based nanoparticles, peptide assemblies, protein cages, exosomes, and DNA origami) (Li et al., 2017). Among these, peptide-based supramolecular nanostructures are an important type of carriers for drug delivery because of the following reasons: (i) the unique biochemical functionality encoded by peptide sequences enables an active targeting (i.e., peptides are targeted to receptors that are overexpressed on cancer cells) or cell membrane–penetrating processes (Wei et al., 2013; Kebebe et al., 2018); (ii) the structure of peptide assembly can be programmatically modulated by intrinsic or/and external stimuli to achieve a controllable release of the payload into the target region (Wei et al., 2013; Raza et al., 2019); (iii) peptides are biocompatible compared to the synthetic organic compounds; (iv) solid-phase synthesis benefits peptide synthesis with the simplicity of operation; (v) the reactive terminus or/and side chains of peptides can be used as a reactive site to conjugate chemotherapeutics (Wyatt et al., 2017; An et al., 2019).

The distinct physical and biochemical characteristics of tumors differing from normal tissues provide promising targets for engineering stimuli–response peptide-assembled DDSs (Ji et al., 2013; Raza et al., 2019; Xiao et al., 2019a; Lian and Ji, 2020). Several hallmarks can be utilized as the triggers to construct stimuli–response DDSs: (i) Acidosis. The overproduction of lactic acid generated by the enhanced glycolysis in tumor cells leads to a slightly acidic tumor microenvironment, i.e., pH 6.5 to 6.8, which is lower than the pH of normal tissues around 7.4 (Romero-Garcia et al., 2011; Ji et al., 2013). (ii) High local temperature. The intrinsic pyrogenic substances secreted by tumor cells induce distinct hyperthermia in the temperature range of 37 to 42°C (Danhier et al., 2010). Typical pyrogenic substances include, inflammatory cytokines, serotonin, catecholamine, and so on. (iii) Redox imbalance. The activation of oncogenes in tumor cells alters the expression and assembly of mitochondrial electron transport chain enzymes and causes a hyperactive reactive oxygen species (ROS) production (Purohit et al., 2019). (iv) Overexpression of certain enzymes such as, the fibroblast-activation protein-α (FAP-α) expressed by cancer-associated fibroblasts (CAFs) (Kalluri and Zeisberg, 2006; Tlsty and Coussens, 2006; Erez et al., 2010; Ji et al., 2013) and matrix metalloproteinases (MMPs) secreted by tumor-associated inflammatory cells in the extracellular matrix (Lu et al., 2012; Ji et al., 2013).

We review recent approaches to the development of stimuli–response self-assembled peptide-based materials that exhibit promising therapeutic effects to deliver drugs to tumor vasculature or tumor cells. The pH, temperature, redox potential, and overexpressed proteins accumulated in tumor cells or tumor microenvironment serve as stimuli to trigger the switch of peptide assemblies. Such sensitive structural conversion of the peptide-assembled delivery systems executes controlling drug release and the intracellular uptake/penetration.

## pH-Responsive Platforms

**Figure 1:**
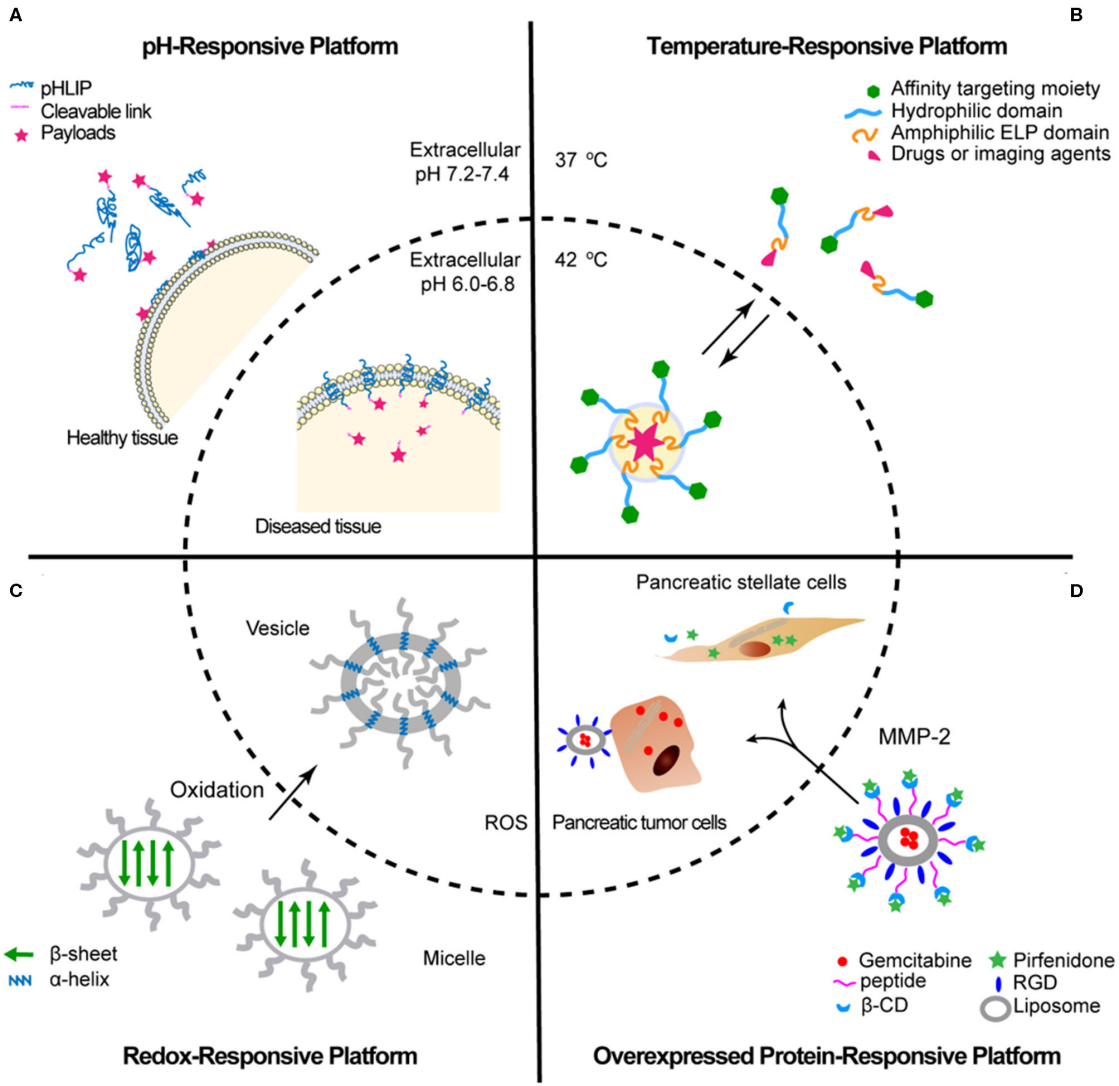
Schematic representations of peptide in response to the acidic pH **(A)**, high temperature **(B)**, the high oxidative potential **(C)**, and the overexpressed protein MMP-2 **(D)**.

Peptides that spontaneously undergo morphological transitions in response to the slightly acidic microenvironments of tumor tissues have the potential to amplify the accumulation of agents used in drug delivery and medical imaging. One method of designing this pH-responsive peptide is using the acidic pH of the solution to toggle the ionic side chains between their charged and neutral states and lead to a structural change driven by the change in charge attractions or repulsions. For example, the pH (low) insertion peptides (pHLIP) derived from the C-helix of a membrane protein bacteriorhodopsin are applied for targeting acidic tissues. The deprotonated side chains of aspartic acid and glutamic acid interspersed throughout the hydrophobic middle region and the C-terminal are negatively charged at physiological pH (pH 7.4), which contributes to an equilibrium between the solvated state and the membrane-attached state of the pHLIP (Reshetnyak et al., 2007). In the acidic environment (pH <6.8), the side chains of aspartic acid and glutamic acid are protonated; the hydrophobicity of pHLIP is increased because the hydration free energy of carboxylic acid (propionic acid, −27 kJ/mol) is less than that of carboxylate (propionate, −331 kJ/mol). The protonated pHLIP inserts into the cell membrane and forms a transmembrane helix. In the membrane-inserted state, pHLIP is oriented with the C-terminus located in the cytosol and the N-terminus exposed to the extracellular space (Wyatt et al., 2018). Cargo molecules can be covalently conjugated with the N-terminus (e.g., fluorescent or radioactive labels) or/and the C-terminus (e.g., translocating cargos) (a cleavable linker is usually needed when conjugating the cargo to the C-terminus). Thus, pHLIP and its derivatives exhibit a wide range of promising applications including intracellular delivery of therapeutic agents (An et al., 2010; Yao et al., 2013; Burns et al., 2015) and fluorescence-guided surgery and imaging (Reshetnyak et al., 2011; Adochite et al., 2014; Tapmeier et al., 2015; Golijanin et al., 2016), as well as diagnostic nuclear imaging (Macholl et al., 2012; Demoin et al., 2016) (Figure 1A). One of the most attractive applications of pHLIP is its ability to facilitate the translocation of cyclic toxins, such as, amanitin, phalloidin, and phallacidin from *Amanita phalloides*, across the lipid bilayer to reach the cytoplasmic targets (An et al., 2010; Wijesinghe et al., 2011). The extracellular delivery of polar cargos expands the drug pipeline and benefits the clinical trial of drug candidates that exhibit promising activity but are too polar by normal drug criteria. Besides pHLIP, the design strategy that the weak acidity of solution switches the charge state of peptides has been implemented to create a series of pH-responsive DDSs, such as, pH-sensitive polyhistidine (PolyHis) (Zhao et al., 2012, 2016) and collagens (Xu et al., 2016; Yang et al., 2017; Yao et al., 2019).

Another strategy to construct pH-responsive peptide DDSs is to introduce an acid-active protecting group into the charged side chains of a peptide. When the peptide is encountering acidic media, the protecting group is removed by acid and converts peptide structure. For example, the positively charged lysine side chains within a cell-penetrating peptide, transactivator of transcription (TAT), can be amidized to be neutral succinyl amides (^a^TAT) (Jin et al., 2013). At physiological pH, the amidized modification of lysine side chains efficiently reduces the non-specific interactions between ^a^TAT and cell membranes. The ^a^TAT-functionalized PEG-PCL micelles (where PEG presents polyethylene glycol, and PCL presents poly ε-caprolactone) loaded with doxorubicin showed a long circulation time in the blood compartment. Once the micelles extravasated from the bloodstream into an acidic environment (tumor tissues or endosomes/lysosomes), the succinyl amides in the ^a^TAT were quickly hydrolyzed (Helmlinger et al., 1997), leading to a fast intracellular uptake/penetration of the nanoparticles.

## Temperature-Responsive Platforms

Hydrophobic interactions, which are encoded by amino acid composition and the non-polar domain size, determine the tertiary and quaternary structure stability and guide the temperature-dependence of peptide assembly (Li and Walker, 2012; Davis et al., 2013). Specifically, the temperature dependence of hydrophobic hydration energy is positive when the size of the non-polar solute is smaller than 1 nm. In contrast, the temperature dependence is negative when the size of the non-polar solute is larger than 1 nm (Li and Walker, 2012; Davis et al., 2013). This analysis provides a theoretical basis for understanding the temperature-triggered assembly structural transition. For example, elastin-like polypeptide (ELP) is a set of commonly used thermally active biopolymers consisting of repeats of VPGXG (where X can be any amino acid except proline) (Urry, 1992). The hydrodynamic diameter of each non-polar patch within an ELP is smaller than 1 nm. Thus, ELPs display a facilitated hydrophobic aggregation upon heating; i.e., a phase transition occurs from clear solution to insoluble aggregate as the temperature increases from below their transition temperature (*T*_t_) to above their *T*_t_ (Urry, 1992; Urry and Pattanaik, 1997; Li et al., 2001). Herein, the *T*_t_ of the polypeptide is a function of residue composition, residue arrangement, biopolymer persistence length, molecular concentration, and coexisted molecules (Meyer and Chilkoti, 2002, 2004). Usually, the *T*_t_ of the polypeptide is designed to be in the temperature range of hyperthermia (ca. 38–42°C).

By using ELP as a skeleton, thermally triggered peptide delivery systems were usually designed as a diblock copolymer consisting of an amphiphilic ELP domain and a hydrophilic domain to covalently tether chemotherapeutic compound (Dreher et al., 2008; McDaniel et al., 2013). In normal tissues, where the physiological temperature is 37°C, chimeric peptides stay in a freely monomeric state. In tumor regions, where the local temperature is >37°C exceeding *T*_t_, the amphiphilic ELP domains assemble to a micelle, and the hydrophilic domains act as linkers to expose the affinity targeting moieties on the outside surface of the micelle (Figure 1B). This feature leads to chimeric peptide–drug complex aggregation and accumulation in tumor tissues by hyperthermia.

## Redox-Responsive Platforms

Excess production of ROS by tumor cells results in pathological stress to cells and tissues, including essential protein damage, lipid peroxidation, DNA strand breakage, and so on (Darley-Usmar and Halliwell, 1996). In terms of protein structure, the thiol groups of cysteine (Cys, C) side chains are attractive oxidative targets for chemical modification by ROS, such as, hydrogen peroxide (H_2_O_2_). The fluctuation in the non-covalent interactions associated with the oxidization of cysteine can lead to the emergence of changed protein tertiary and quaternary structures. This observation motivates researchers to design redox-stimulated peptide assemblies by using cysteine or cysteine derivative as a ROS sensor. In their systems, a change in the intermolecular interactions encoded by cysteine, which results from the presence of ROS, is amplified into a range of structural outputs. For instance, cholesterol-decorated peptide PEG-PCys-Chol adopted an antiparallel β-sheet conformation and assembled into micelles in solution (where PEG presents polyethylene glycol, PCys presents polyC, and Chol presents cholesterol) (Liu et al., 2018). After treatment with 10% H_2_O_2_ in the presence of 5% acetic, the side chains of polyC were oxidized. The oxidation of PEG-PCYs-Chol (PEG-PCys-Chol-O_2_) adopted a helical conformation and displayed a micelle-to-vesicle structural transformation (Figure 1C). The oxidized PEG-PCys-Chol-O_2_ vesicle exhibited a strong potency to deliver a model chemotherapeutic agent doxorubicin (DOX) into HeLa tumor cells. In contrast, HeLa cells were inert to the reductive PEG-PCys-Chol/DOX micelle. The high internalization efficiency of PEG-PCys-Chol-O_2_/DOX is due to the cholesterol-bearing α-helical structure that facilitates cell membrane penetration (Kulkarni et al., 2012; Yin et al., 2013; Sevimli et al., 2015). Collectively, this effort demonstrates a strategy of triggering a controllable release of cargos by hyperactive ROS in tumor tissues.

## Overexpressed Protein-Responsive Platforms

Overexpressed proteins within tumor tissues provide specific binding targets for recruiting drug/delivery systems to improve the bioavailability of drugs (Kalluri and Zeisberg, 2006; Tlsty and Coussens, 2006; Erez et al., 2010; Sagnella et al., 2014). Biocatalysis-dependent delivery systems that target these characteristic proteins were delicately designed. The system usually contains two functional moieties: a sensor moiety that acts as an enzyme substrate and a cohesion moiety that drives peptide to assembly. Thus, the process of biocatalysis-dependent recognition involves an enzyme-mediated change with peptide assembly structures.

For example, FAP-α is selectively overexpressed by CAFs, the predominant cell type in the tumor stroma. Several stimuli-responsive nanostructures for drug delivery and release have been constructed inspired by the catalytic function of FAP-α. Ji et al. (2016b) designed a biocatalytic amphiphilic peptide Ac-ATK(C_18_)DATGPAK(C_18_)TA-NH_2_ (where C_18_ represents an octadecanoic acid chain) that exhibited morphological changes mediated by FAP-α. The moiety of -GPAX- is a specific cleavage substrate of FAP-α, and the moieties of -ATK(C_18_)- and -(C_18_)TA- provide hydrophobic attractions for peptide assembly (Ji et al., 2016b). In water environment, hydrophobic drug Dox coassembled with Ac-ATK(C_18_)DATGPAK(C_18_)TA-NH_2_ to form spherical nanoparticles. When interacting with CAFs, nanoparticles disassembled rapidly under FAP-α's cleavage and efficiently released DOX specifically at the tumor sites. Thus, the peptide assemblies could enhance the drug perfusion in solid tumor treatment.

Similarly, the diverse chemokines, cytokines, and matrix-degrading enzymes secreted by tumor-associated inflammatory cells, such as, MMPs, are also promising targets for the design of an enzyme-active peptide-based delivery system. Ji et al. (2016a) developed an MMP-2–sensitive peptide linker CSSSGPLG-IAGQSSS to tether (i) a gemcitabine/RGD (tumor cell–binding peptide) loaded liposome and (ii) a pirfenidone-loaded β-cyclodextrin (β-CD) (Figure 1D). This peptide–β-CD–liposome supramolecular architecture was cleaved into two active units when they reached the tumor tissues to exert a synergy effect against pancreatic tumor: (i) The gemcitabine/RGD-loaded liposome unit targeted and directly killed pancreatic tumor cells. (ii) The β-CD–pirfenidone unit was maintained in the tumor stroma to down-regulate the fibrosis and decrease the stromal barrier.

## Conclusion and Challenges

In summary, we present a brief overview of the strategies available to the rational design of peptide-assembled agent delivery nanoplatforms. The architectures of peptide-based DDSs are precisely engineered to achieve active drug transportations and release upon tumor microenvironment stimuli, including pH, temperature, redox potential, and tumor-associated overexpressed proteins. Despite the promising activity, there still exist challenges that limit the application of stimuli-responsive peptide-based nanomaterials in tumor treatment.

(i) High interstitial fluid pressure in most solid tumors impedes the penetration of compounds and delivery systems (Heldin et al., 2004). The reasons for increased interstitial fluid pressure probably involve blood-vessel leakiness, lymph-vessel abnormalities, interstitial fibrosis, and a contraction of the interstitial space mediated by stromal fibroblasts. High-molecular-weight compounds, such as, peptide assemblies, are transported in the interstitium mainly via convection rather than via diffusion (Heldin et al., 2004; An et al., 2019). Thus, a high interstitial fluid pressure induces a reduction of transcapillary transport and weakens the streaming of therapeutic drugs and delivery platforms from the circulation system through the interstitial space. Some of the cytokine antagonists, such as, vascular endothelial cell growth factor antagonist and platelet-derived growth factor (PDGF) antagonist, can be applied to lower tumor interstitial fluid pressure and facilitate the cellular uptake of drugs. However, it is still a challenge to reduce the interstitial fluid pressure of tumors without affecting normal tissues (Heldin et al., 2004). In addition, drugs or drug-loaded supramolecules that self-assemble *in situ* and exhibit high retention efficiency in the specific tumor tissues also provide a promising solution for overcoming the high interstitial fluid pressure obstacle to adequate drug delivery (Gao et al., 2013; An et al., 2019), but these studies are still in the preliminary stage of exploration.

(ii) Advanced methodologies and technologies are required to track the fate of stimuli-responsive peptide-based DDSs *in vivo* (Kreyling et al., 2015; Chen et al., 2017). Recent *in vivo* studies with a few numbers of nanosystems indicate that the surface chemistry, the corona of adsorbed proteins, and integrity of assembly nanostructures can be dramatically degraded by enzymes and immune cells and consequently change the pharmacokinetics, biodistribution, and immunological effects of drug carriers (Kreyling et al., 2015; Chen et al., 2017). It is important to investigate whether the stimuli response of peptide-based DDSs *in vitro* can be manifested *in vivo*. However, such measurement is challenging because of the lack of a feasible analytical instrument or methodology to reveal the structural transitions of peptide-based DDSs *in vivo*. Inspired by other nanomedicine research (Shan et al., 2018, 2019), some potential strategies are expected to solve this thorny problem, including high performance liquid chromatography measurement of plasma level of the loaded drugs, detection of specific overexpressing proteins/micromoles in the blood, and development of harmless tracer/probe. This research direction needs more efforts in the future.

(iii) The clinical evaluation of the stimuli-responsive peptide DDSs is absent because of the expensive and lengthy regulatory process (Jang et al., 2016). The behaviors of DDSs in human bodies might deviate from that in animal models as they possess different inherited characteristics (Jang et al., 2016). Innovative preclinical testing platforms, such as, patient-derived organoids, are needed to accurately evaluate the efficacy and safety of stimuli-responsive peptide-based DDSs before clinical trials (Tiriac et al., 2018; Saito et al., 2019).

Efforts to address the aforementioned challenges will substantially promote the medical application of stimuli–response peptide-assembled nanomaterials as a chemotherapeutics delivery platform and finally benefit cancer treatment in the clinic.

## Author Contributions

All authors listed have made a substantial, direct and intellectual contribution to the work, and approved it for publication.

## Conflict of Interest

The authors declare that the research was conducted in the absence of any commercial or financial relationships that could be construed as a potential conflict of interest.
